# Naringenin: A Promising Therapeutic Agent against Organ Fibrosis

**DOI:** 10.1155/2021/1210675

**Published:** 2021-11-11

**Authors:** Yanfei Du, Jun Ma, Yu Fan, Xinyu Wang, Shuzhan Zheng, Jian Feng, Jiafu Li, Zhongcai Fan, Guang Li, Qiang Ye

**Affiliations:** ^1^Department of Cardiology, The Affiliated Hospital of Southwest Medical University, Key Laboratory of Medical Electrophysiology of Ministry of Education and Medical Electrophysiological Key Laboratory of Sichuan Province, Collaborative Innovation Center for Prevention and Treatment of Cardiovascular Disease, Institute of Cardiovascular Research, Southwest Medical University, Luzhou, Sichuan 646000, China; ^2^Department of Obstetric, The Affiliated Hospital of Southwest Medical University, Luzhou, Sichuan 646000, China; ^3^Key Laboratory of Medical Electrophysiology of Ministry of Education and Medical Electrophysiological Key Laboratory of Sichuan Province, Collaborative Innovation Center for Prevention and Treatment of Cardiovascular Disease, Institute of Cardiovascular Research, Southwest Medical University, Luzhou, Sichuan 646000, China

## Abstract

Fibrosis is the final common pathology of most chronic diseases as seen in the heart, liver, lung, kidney, and skin and contributes to nearly half of death in the developed countries. Fibrosis, or scarring, is mainly characterized by the transdifferentiation of fibroblasts into myofibroblasts and the excessive accumulation of extracellular matrix (ECM) secreted by myofibroblasts. Despite immense efforts made in the field of organ fibrosis over the past decades and considerable understanding of the occurrence and development of fibrosis gained, there is still lack of an effective treatment for fibrotic diseases. Therefore, identifying a new therapeutic strategy against organ fibrosis is an unmet clinical need. Naringenin, a flavonoid that occurs naturally in citrus fruits, has been found to confer a wide range of pharmacological effects including antioxidant, anti-inflammatory, and anticancer benefits and thus potentially exerting preventive and curative effects on numerous diseases. In addition, emerging evidence has revealed that naringenin can prevent the pathogenesis of fibrosis in vivo and in vitro via the regulation of various pathways that involved signaling molecules such as transforming growth factor-*β*1/small mother against decapentaplegic protein 3 (TGF-*β*1/Smad3), mitogen-activated protein kinase (MAPK), phosphatidylinositol 3-kinase/protein kinase B (PI3K/Akt), sirtuin1 (SIRT1), nuclear factor-kappa B (NF-*κ*B), or reactive oxygen species (ROS). Targeting these profibrotic pathways by naringenin could potentially become a novel therapeutic approach for the management of fibrotic disorders. In this review, we present a comprehensive summary of the antifibrotic roles of naringenin in vivo and in vitro and their underlying mechanisms of action. As a food derived compound, naringenin may serve as a promising drug candidate for the treatment of fibrotic disorders.

## 1. Introduction

Currently, the incidence of fibrotic diseases is on the rise and presents a serious threat to global public health [1]. Nearly 45% of disease-related deaths in the developed countries are closely associated with fibrotic disorders, and the morbidity and mortality of these disorders are probably higher in the developing countries [2, 3]. Despite much progress made in uncovering the molecular mechanisms underlying the development and progression of fibrosis over the past decades, there is currently no effective antifibrotic treatment available for fibrotic diseases. Therefore, identification of new molecular mechanisms involved in the fibrotic process and development of novel therapeutic agents against fibrotic disorders are urgently needed.

Recently, increasing evidence has demonstrated that many natural products such as flavonoids have potent antifibrotic activities, and some of which have shown promise as emerging new antifibrotic agents [4]. Naringenin, a natural citrus flavonoid that possesses various biological properties, has been extensively reported to prevent the pathogenesis of fibrosis in several experimental studies [5–8]. The importance of naringenin in managing tissue fibrosis warrants a detailed review of the effects of naringenin on fibrosis and of its underlying mechanisms of action. Here, we will focus on the crucial role of naringenin in the suppression of tissue fibrosis and discuss its therapeutic potential as a promising agent for the treatment of fibrotic disorders.

## 2. The Cellular and Molecular Mechanisms of Fibrosis

Fibrosis often takes place in response to a trigger or tissue injury [9]. Initially, tissue injury is mild or transient, and the formation of fibrotic scars in organs is actually a normal tissue repair response and is beneficial for organisms. However, when the injury is severe or prolonged, sustained or uncontrolled fibrogenesis can result in adverse architectural remodeling, organ malfunction, and eventually organ failure. These defects contribute significantly to global morbidity and mortality [10].

**Figure 1:**
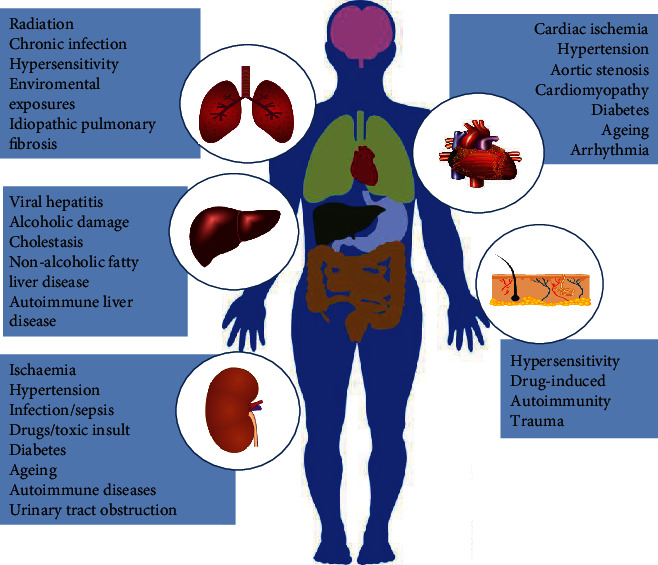
Major causes of organ fibrosis. In the different organs, a broad range of triggers and etiologies can result in occurrence and development of fibrosis. Fibrosis may lead to organ dysfunction or failure and accounts for substantial morbidity and mortality [adapted from ref. [10]].

Fibrosis is the final common pathology of many chronic inflammatory diseases as detected in the heart, kidney, liver, lung, and skin tissue [11]. The causative factors of fibrosis are diverse in the various organs (Figure 1), but the most common etiologies include inflammation, aging, and genetic alteration [4, 12]. Fibrosis is mainly defined as the activation and proliferation of fibroblasts, the production of inflammatory factors, and the massive deposition of extracellular matrix (ECM) proteins such as type I collagen (COL1), type III collagen (COL3), and fibronectin (FN) [13, 14]. Myofibroblasts, the activated form of fibroblasts, exhibit two unique characteristics: firstly, they are contractile due to the expression of *α*-smooth muscle actin (*α*-SMA), which results in the distortion of tissue cytoarchitecture. Secondly, they secrete ECM macromolecules, which lead to the replacement of normal tissue with a permanent fibrotic scar, thus causing an increase in tissue stiffness and the parenchymal destruction of organs [2, 15].

**Figure 2:**
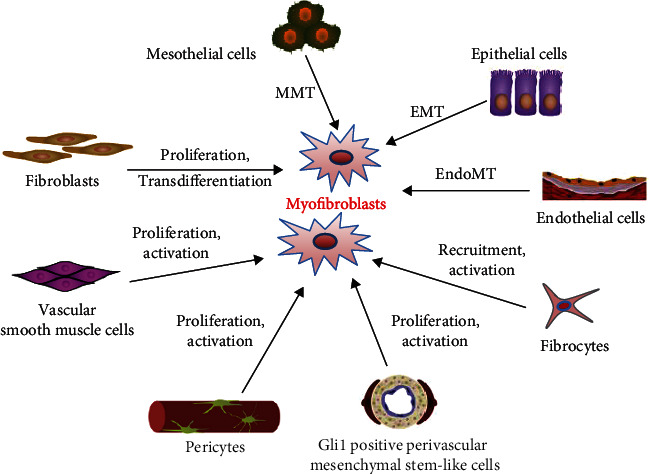
Potential sources and formation mechanisms of myofibroblasts. Activated myofibroblasts are central drivers for fibrosis and can secrete excess extracelluar matrix proteins. The cellular subsets may be originated from resident fibroblasts, epithelial cells, endothelial cells, circulating fibrocytes, mesothelial cells, vascular smooth muscle cells, pericytes, Gli1 positive perivascular mesenchymal stem-like cells, and others. Diverse mechanisms comprising cellular proliferation, activation, transdifferentiation, recruitment, mesothelial-to-mesenchymal transition (MMT), epithelial-to-mesenchymal transition (EMT), and endothelial-to-mesenchymal transition (EndoMT) can lead to myofibroblast formation [adapted from refs. [11, 15]].

The sources of myofibroblasts may vary across different tissues, depending on the injured organ and the specific fibrotic response [16–18]. Several potential sources and formation mechanisms of myofibroblasts are presented in Figure 2. Although epithelial/endothelial-to-mesenchymal transition (EMT/EndoMT), or pericyte to myofibroblast transition may play a role under special conditions, it is now widely accepted that the main source of myofibroblasts is the activation of tissue-resident fibroblasts [16, 18–20].

**Figure 3:**
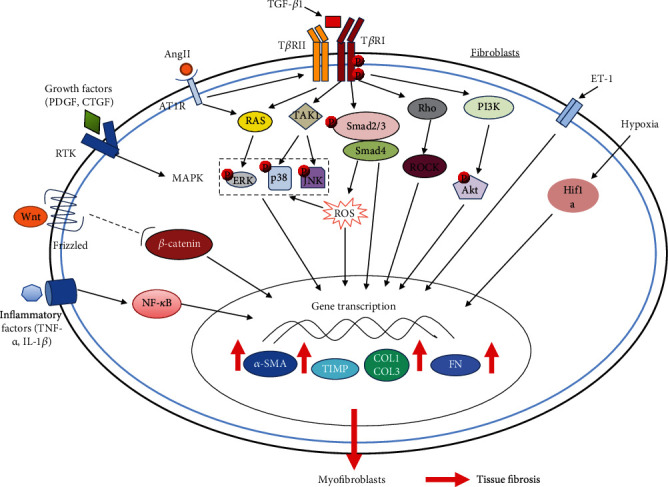
Molecular mechanisms in tissue fibrosis. The diagram shows the TGF-*β*1, AngII, ET-1, growth factors (PDGF, CTGF, etc.), inflammatory factors (TNF-*α*, IL-1*β*, etc.), Wnt, ROS, and hypoxia-inducible factor-1*α* (HIF-1*α*) pathways that may mediate tissue fibrotic responses. The central pathways for tissue fibrosis are TGF-*β*1 canonical (Smad-dependent) and noncanonical (Smad-independent) signaling pathways, among which the canonical TGF-*β*1/Smad pathway plays a major role in the development of fibrosis. Following TGF-*β*1 binding, type II TGF-*β*1 receptor (T*β*RII) recruits type I TGF-*β*1 receptor (T*β*RI) and activates it by phosphorylating it. The activated T*β*RI then specifically phosphorylates Smad2 and Smad3, which then bind to Smad4 to form a complex leading to their translocation to the nucleus and regulation of transcription of profibrotic genes. Apart from Smad-mediated signal transduction, TGF-*β*1 can also signal through several noncanonical signaling cascades such as PI3K, p38, ERK, JNK, and Rho-like GTPase pathways. Most of the other pathways have been indicated to regulate or to interact with the TGF-*β*1 signaling pathways. The final result of these signaling pathways activation is triggering a profibrotic gene transcriptional regulation program contributing to tissue fibrosis caused by the activation of myofibroblasts and their increased synthesis of various myofibroblast-specific and profibrotic proteins such as *α*-SMA, COL1, COL3, FN, and tissue inhibitors of metalloproteinase (TIMP) [Adapted from ref. [15]].

Despite the highly complex mechanisms for fibrosis, the transdifferentiation of fibroblasts into myofibroblasts is a central driver for all forms of fibrosis [10, 15]. To date, a wide range of mediators have been found to activate fibroblasts and to promote the initiation and progression of fibrosis, including transforming growth factor-*β*1 (TGF-*β*1) [21, 22], angiotensin II (AngII) [23, 24], connective tissue growth factor (CTGF) [25, 26], platelet-derived growth factor (PDGF) [27, 28], interleukins (IL-6, IL-13, IL-33, IL-11, IL-17, etc.) [11, 12, 29], tumor necrosis factor-*α* (TNF-*α*) [30], endothelin-1(ET-1) [31, 32], reactive oxygen species (ROS) [33, 34], and hypoxia [35, 36]. Some of these mediators inducing fibrotic processes are illustrated in Figure 3. Among them, TGF-*β*1 is considered to be the most potent profibrotic factor and contributes to fibrosis primarily by activating its downstream canonical small mother against decapentaplegic (Smad) signaling pathway [37]. In addition, TGF-*β*1 has also been shown to act through several Smad-independent pathways (known as noncanonical signaling cascades) in the development of fibrosis, such as mitogen-activated protein kinase (MAPK) pathways mediated by extracellular signal-regulated kinase (ERK), c-Jun N-terminal kinase (JNK), and p38 MAPK as well as phosphatidylinositol 3-kinase/protein kinase B (PI3K/Akt) or Rho-like GTPases signaling pathways [38, 39]. Besides the TGF-*β* signaling pathway, there are numerous other signaling cascades that are also involved in the pathogenesis of fibrosis, such as nuclear receptors signaling (peroxisome proliferation-activated receptor-*γ*, PPAR-*γ*) [9], bone morphogenetic protein (BMP) signaling [40], Wnt/*β*-catenin signaling [41], Hedgehog signaling [42], Notch signaling [43], and epidermal growth factor receptor (EGFR) signaling [44]. Therefore, targeting these fibrotic mediators or signaling pathways could represent potential therapeutic strategies to combating fibrotic diseases.

## 3. Naringenin

**Figure 4:**
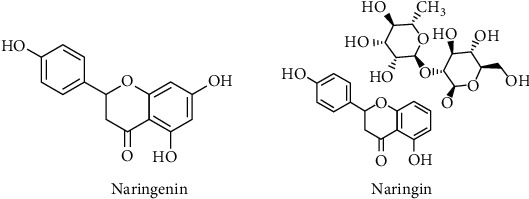
Chemical structures of naringenin and its glycosylated form naringin.

Naringenin, 5,7-Dihydroxy-2-(4-hydroxyphenyl) chroman-4-one, is one of the most important natural flavonoids and mostly exists in citrus fruits like grape fruits, orange and lemon [45, 46]. It has a molecular weight of 272.26 (C_15_H_12_O_5_) and exists predominantly in nature in two forms: the glycosylated form (naringin or naringenin-7-O-glucoside) and the aglycosylated form (naringenin) (Figure 4) [47]. Naringin can be hydrolyzed into naringenin by the liver enzyme naringinase [48], and naringin is responsible for the bitter taste of citrus fruits, whereas naringenin is flavorless.

In nature, naringenin exists as a solid and is almost insoluble in water, but soluble in organic solvents such as dimethyl sulfoxide and ethanol. However, naringin can easily dissolve in water [49]. Although naringenin is quickly absorbed after its single oral administration in human subjects, it shows only 5.81% oral bioavailability due to its poor aqueous solubility, which compromises its clinical use [50]. Both active transport and passive diffusion aid its absorption into the gastrointestinal tract [45]. After absorption, naringenin is rapidly conjugated to form glucuronide or sulphoglucuronide and is bound to serum albumin and is rapidly transported to highly perfused organs like the kidney, heart, spleen, liver, and cerebrum [51, 52]. Before absorption, naringenin is hydrolyzed by *β*-glucosidase in the small intestine [53, 54]. Then, naringenin is further metabolized by the intestinal bacteria into p-hydroxyphenylpropionic acid, phenolic acids, and p-hydroxybenzoic acid which are mainly found in the plasm, urine, and bile [55, 56]. The excretion of naringenin occurs through two primary pathways: urinary and biliary pathways [57]. It is suggested that naringenin has low toxicity, and its LD50 is 5000 mg/kg [58]. Despite the fact that naringenin has relatively low bioavailability, there are currently some techniques and pharmacological formulations to improve its bioavailability, such as developing drug delivery systems by the use of liposomes, nanoparticles, nanosuspensions, solid dispersion, and inclusion complexation [45, 59].

In various in vitro and in vivo studies, naringenin has been demonstrated to exhibit extensive biological activities, including antioxidant [60, 61], anti-inflammatory [62, 63], antiviral [64, 65], antibacterial [66, 67], and anticancer actions [68, 69]. Owing to these pharmacological properties, naringenin has been reported to exert a strong protective role and has therapeutic potential against numerous diseases, like cardiovascular diseases [55, 70], liver diseases [71], lung diseases [72], diabetes [58, 73], neurodegenerative diseases [74, 75], and malignant tumors [76, 77]. In addition, recently, emerging evidence has suggested that naringenin is capable of inhibiting the progression of fibrosis in multiple organs and tissues, including the liver, heart, lung, kidney, and skin, through regulation of various signaling pathways (Table 1) and thus showing potential therapeutic effects on fibrotic disorders [8].

## 4. Naringenin and Organ Fibrosis

### 4.1. Naringenin and Liver Fibrosis

Liver fibrosis is a reversible wound-healing response to acute or chronic liver injury from various causative factors, like chronic viral infection, excess alcohol consumption, toxic exposure, cholestasis, autoimmune hepatitis, and nonalcoholic steatohepatitis (NASH) [78, 79]. Hepatic fibrosis, mainly characterized by the excessive accumulation of ECM protein and the formation of fibrous scar in injured liver [80], is the final common pathology of all chronic liver diseases. It can ultimately lead to irreversible liver cirrhosis, which is the end stage of liver disease and also one of the most common causes of morbidity and mortality worldwide [81, 82]. Thus, prevention and reversal of hepatic fibrosis are an effective strategy for treating various chronic liver diseases and combating cirrhosis. However, up to now, there is no effective therapeutic treatment for liver fibrosis except for the removal of the causative factor or liver transplantation [80]. Hepatic stellate cells (HSCs) are the central effectors in the development of liver fibrosis, which are the primary source of abnormal ECM constituents in the liver [83, 84]. Upon fibrogenic stimulation, such as exposure to injury or profibrotic factor, HSCs become activated and begin to over proliferate and transdifferentiate into myofibroblasts, which massively express *α*-SMA and ECM proteins, thereby leading to liver fibrogenesis [85, 86]. Therefore, targeting HSC activation and proliferation has been considered as a promising therapeutic strategy for the treatment of liver fibrosis.

There have been now many studies indicating the therapeutic roles of naringenin in preclinical models of liver fibrosis. Lee et al. first suggested some histological evidence that oral administration of naringenin could reduce hepatic collagen accumulation and exert potential antifibrotic effects in rats with liver damage induced by dimethylnitrosamine (DMN) via the inactivation of HSCs [6]. In an in vitro study, naringenin was for the first time demonstrated to be a Smad3 specific inhibitor and could suppress the TGF-*β*1-induced ECM protein expression in cultured rat HSCs by blocking the TGF-*β*1 signaling pathway via selectively inhibition of Smad3 activation [87]. In a rat model of high cholesterol-induced hepatic damage, naringenin supplementation alleviated hepatic oxidative stress and inflammatory response, as well as collagen deposition as indicated by Sirius Red staining of liver sections, by inhibiting NF-*κ*B pathway and matrix metalloproteinases-2/9 (MMP-2/9) activities, respectively, ultimately attenuating fibrosis and the liver injury [88]. In addition, in another rat model of liver fibrosis, Hernández-Aquino et al. showed that naringenin was able to block carbon tetrachloride- (CCl_4_-) induced liver inflammation, necrosis, and fibrosis by reducing oxidative stress as well as by preventing NF-*κ*B, TGF-*β*/Smad3, and JNK/Smad3 signaling pathways [89], which was in agreement with the findings reported by the same research team in another study [90]. In a mouse model of chronic alcohol-induced hepatic damage, Zhang et al. demonstrated that naringenin treatment could prevent hepatic inflammation, suppress liver fibrosis, and alleviate hepatocyte apoptosis, thus improving the liver function, through decreasing the levels of NF-*κ*B, TGF-*β*1, and caspase-3, respectively [91]. In another report, to increase the bioavailability and HSCs-targeted property of naringenin, Wang et al. developed a novel activated HSCs-targeted drug delivery system, namely, naringenin-loaded albumin self-modified liposomes (NaAlLs), and demonstrated that NaAlLs significantly, and specifically, increased targeting of activated HSCs and ameliorated liver fibrosis in vitro and in vivo via the secreted protein acidic and rich in cysteine- (SPARC-) dependent pathway [92]. Moreover, in a mouse model of NASH, naringenin administration could suppress hepatic steatosis, reduced hepatic oxidative stress and inflammation, and prevented liver fibrosis, as evidenced by the decrease in hepatic collagen deposition and hydroxyproline content, as well as by the reduction of protein expression of TGF-*β*1 and *α*-SMA in the liver. This process was mediated by the activation of hepatic sirtuin1- (SIRT1-) mediated signaling cascades that led to the therapeutic effects of naringenin on NASH [93]. In a recent report, Yang et al. discovered that naringenin loaded nanoparticles, which could enhance the oral bioavailability of naringenin, markedly reduced CCl_4_-induced liver fibrosis and inflammation in rats, as assessed by liver histology and serum levels of inflammatory cytokines, via upregulating the activity of MMP-2 and decreasing the levels of proinflammatory cytokines [94].

### 4.2. Naringenin and Cardiac Fibrosis

Cardiac fibrosis is a final pathological outcome for multiple forms of cardiovascular diseases, including cardiomyopathy, hypertension, arrhythmias, and myocardial infarction [10]. It represents a substantial accumulation of ECM proteins in the interstitium of the heart and excessive cardiac scar formation, which causes electrical and mechanical dysfunction, thereby ultimately contributing to heart failure and death [95, 96]. The risk factors of myocardial fibrosis are diverse, and some of the common ones are described in Figure 1. Cardiac fibroblasts (CFs) are the predominant cell type within the myocardium that provides structural support [97]. Upon injury or stimuli, CFs can proliferate abnormally and transdifferentiate into activated myofibroblasts, which is the key event in cardiac fibrosis. Despite extensive research, the underlying mechanisms of cardiac fibrosis are not fully elucidated and currently, no evidence-based therapies show significant effectiveness on treating cardiac fibrosis.

The first direct evidence of an interaction between naringenin and cardiac fibrosis was that naringenin was shown to alleviate pressure overload-induced cardiac hypertrophy and interstitial fibrosis in mice, as assessed by histological analysis and quantitative PCR analysis of hypertrophy biomarkers and profibrotic genes [70]. The potential mechanisms of naringenin exerting its cardioprotective effect may be related to the suppression of ERK, JNK, and PI3K/Akt signaling pathways. An in vitro study by Liu et al. reported that naringenin was able to inhibit TGF-*β*1-induced proliferation, transformation, and collagen production of CFs, and the mechanism underlying the process may be in part due to the inhibition of DNA synthesis via G0/G1 arrest following treatment with naringenin, thus implying that naringenin may serve as a novel treatment strategy for cardiac fibrosis [98]. In addition, Wei et al. found that naringenin, as a Smad3-specific inhibitor, could attenuate hypertension-induced atrial fibrosis in spontaneously hypertensive rats (SHRs) and inhibit the proliferation and ECM protein expression of CFs induced by elevated hydrostatic pressure via the suppression of Smad3 signaling activation [7]. In a recent report, Liang et al. suggested that naringenin significantly inhibited the protein expression of profibrotic genes such as COL1, COL3, and ACTA2 (actin alpha 2, smooth muscle) through inactivating the Smad3 signaling pathway in AngII-stimulated mouse CFs [99], also revealing the potential of naringenin to treat cardiac fibrosis.

### 4.3. Naringenin and Lung Fibrosis

Pulmonary fibrosis refers to the end stage of various interstitial lung diseases, characterized by phenotypic alteration of both fibroblasts and alveolar epithelial cells, abnormal deposition of ECM, and the disruption of lung parenchyma, which results in impaired gas exchange, decreased lung function, and progressive respiratory failure [100]. So far, a variety of underlying etiologies have been identified to lead to lung fibrosis, such as ageing, environmental and occupational exposures, autoimmune diseases, and genetic disorders; yet, the most common form is idiopathic pulmonary fibrosis (IPF) [101]. IPF is a progressive and terminal lung disease with 3-5 years of median survival time after diagnosis. The incidence of this disease has risen. Currently, two small-molecule drugs, pirfenidone [102], and nintedanib [103], which have been demonstrated to slow disease progression, have been approved worldwide for the treatment of IPF; however, they have toxic side-effects and cannot reverse fibrosis [102, 103]. As such, for now, lung transplantation is the sole therapeutic strategy.

Studies have shown that naringenin exhibits antifibrotic effects, as a potential drug to treat pulmonary fibrosis that arises from various etiologies. In a mouse model of bleomycin-induced pulmonary fibrosis, Du et al. demonstrated that oral administration of naringenin attenuated bleomycin-induced pulmonary fibrosis, as shown by histological staining and quantification of collagen content in the lung, by inhibiting TGF-*β*1 secretion and decreasing regulatory T cells [5]. In a murine model of asthma, Shi et al. suggested that naringenin treatment inhibited allergen-induced airway remodeling and peribronchial fibrosis as evidenced by the decreases in peribronchial *α*-SMA areas, subepithelial collagen deposition, and hydroxyproline content in the lung, probably through reducing T-helper 2 (Th2) cytokine levels and oxidative stress [104]. Similarly, in another murine asthma model induced by house dust mite (HDM), Seyedrezazadeh et al. found that a combination of naringenin with other flavanone, hesperetin, could markedly alleviate HDM-induced airway inflammation and fibrosis, as assessed by histological analysis, potentially through interfering with the expression of proinflammatory cytokines and TGF-*β* [105]. In a study of *mycoplasma pneumoniae* (MP) pneumonia, Lin et al. identified that treatment with naringenin could suppress MP-induced lung inflammation and fibrosis in vivo and also suppressed MP-induced BEAS-2B cell injury in vitro, by inhibition of the autophagy pathway [106]. In addition, in rodent models of radiation-induced lung injury, Zhang et al. proved that naringenin treatment effectively ameliorated radiation-induced lung injury, including lung fibrosis as assessed by histological analysis, by lowering IL-1*β* and maintaining the homeostasis of inflammatory factors [107].

### 4.4. Naringenin and Renal Fibrosis

Chronic kidney diseases (CKD), with a high prevalence of morbidity and mortality, remain a major global public health problem imposing enormous economic burden on society [108, 109]. Renal fibrosis is the common final pathway of almost all progressive CKD with diverse etiologies (including ischaemia, infection, autoimmune disease, toxic/drug insult, diabetes, and genetic disorders), and it has been indicated to be the best predictor of CKD progression to end-stage renal disease, which requires dialysis or kidney transplantation [110, 111]. Renal fibrosis is typically marked by infiltration of inflammatory cells and activation and proliferation of myofibroblasts, which leads to excessive accumulation of ECM components in the glomeruli, interstitium, and vasculature. Currently, there are no specific antifibrotic drugs in use for kidney patients [112]. Therefore, development of effective therapeutic treatments to treat kidney fibrosis is of utmost importance.

A previous study showed that treatment with naringenin could improve daunorubicin-induced nephrotoxicity in rats by reducing renal fibrosis, inflammation, and oxidative/endoplasmic reticulum stress, which may be possibly through the mitigation of AngII type I receptor (AT1R), ERK1/2-NF-*κ*B p65 signaling pathways [113]. In addition, Meng et al. demonstrated that only naringenin treatment markedly alleviated renal fibrosis in vitro and in a mouse model of unilateral ureteral obstruction (UUO) by blocking Smad3 signaling directly, and the combination of naringenin with asiatic acid, a triterpene from Centella Asiatica, demonstrated to be a Smad7 agonist, produced a better inhibitory effect on renal fibrosis by suppressing Smad3 while inducing Smad7 [114]. In a study of diabetic nephropathy (DN), Yan et al. suggested that naringenin could inhibit the expressions of ECM components in both kidney tissues of DN rats and glomerular mesangial cells treated by high glucose and also inhibited mesangial cell proliferation, by suppressing TGF-*β*1/Smad signaling pathway via the regulation of microRNA let-7a [115]. In an animal model of renovascular hypertension established by performing 2-kidney, 1-clip surgery in rats, Wang et al. observed that naringenin administration significantly ameliorated hypertensive renal damage in the nonclipped kidneys, including interstitial fibrosis as measured by histological analysis, by normalizing the imbalance of renin-angiotensin system [116]. Moreover, naringenin was identified to prevent autoimmune features and kidney injury, including renal fibrosis as evaluated by the decrease in collagen fibers, in lupus-prone mice, by modulating T-cell subsets and cytokines profile [117].

### 4.5. Naringenin and Skin Fibrosis

Skin fibrosis, as defined by excessive fibroblast proliferation and ECM protein deposition in the dermis, is the common pathological hallmark of multiple skin disorders such as systemic sclerosis, hypertrophic scars, keloids, restrictive dermopathy, and graft-versus-host disease [118]. Skin fibrosis affects over 100 million people per year in westernized countries and becomes a significant health problem worldwide [119, 120]. Cutaneous scars have a profound impact on patients' quality of life due to related pain and pruritus, functional impairment, and psychosocial distress [119, 121, 122]. Despite the socioeconomic burden, effective and durable scar treatment remains a major unmet need in clinical medicine [123].

In a mouse model of mechanical stretch-induced hypertrophic scars, topical application of naringenin could attenuate skin fibrosis and inhibit scar formation, as assessed by histological analysis, by the suppression of dermal fibroblast activation and local inflammatory response, thus implying that naringenin may serve as a novel agent for treating hypertrophic scars [124]. As there are few reports about the effect of naringenin on skin fibrosis, the antifibrotic role of naringenin in skin tissues remains to be further elucidated.

## 5. Conclusions and Perspectives

**Figure 5:**
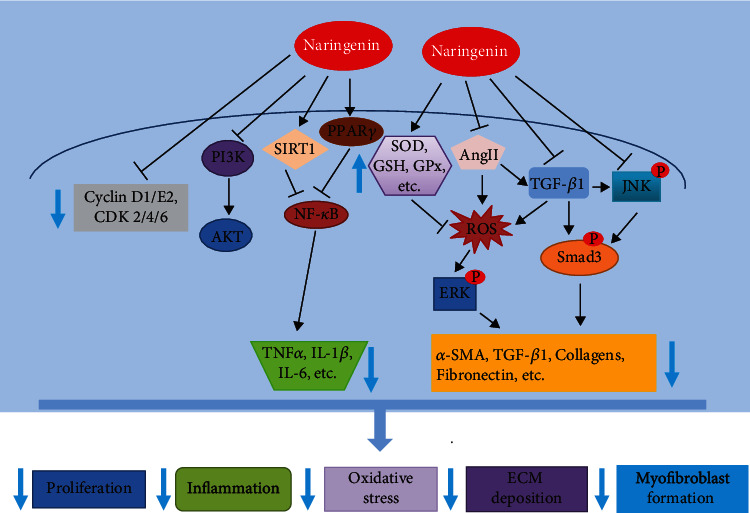
Antifibrotic mechanisms of naringenin: schematic representation of naringenin exerting its antifibrotic effects through affecting multiple signaling pathways related to fibrogenesis (↑: increase; ↓: decrease; SOD: superoxide dismutase; GSH: glutathione; GPx: glutathione peroxidase; CDK: cyclin-dependent kinases).

In this review, we summarize the recent advances of naringenin in fibrosis research and treatment. A growing body of evidence, both in vitro and in vivo, has indicated that naringenin exerts potential antifibrotic properties in multiple tissues and organs like the liver, heart, lung, kidney, and skin, and their mechanisms of action, which have been summarized in Figure 5, may involve mostly the regulation of TGF-*β*1/Smad3, MAPK, PI3K/Akt, SIRT1, and NF-*κ*B signaling pathways, as well as oxidative stress. However, the antifibrotic effects of naringenin are mostly derived from animal studies and cellular models of fibrosis, and there is a lack of clinical trial evidence. In addition, the antifibrotic mechanisms of naringenin have not been fully delineated. The studies regarding the safety and efficacy of naringenin in humans are still lacking. Going forward, more mechanistic and clinical studies are needed to further support the utilization of this flavonoid in human diseases. Despite that naringenin has very low water solubility, which leads to its low bioavailability, there are some techniques and methods to solve this problem, such as designing an oral drug delivery system using liposomes, nanoparticles, or nanosuspensions [45, 59]. In summary, as a food-derived compound, naringenin may serve as a promising therapeutic agent for fibrotic disorders in the future.

## Figures and Tables

**Table 1:** Summary of preclinical antifibrotic effects and underlying mechanisms of naringenin.

Fibrotic disease	Models	In vitro/in vivo	Effects and related mechanisms	Reference
Liver fibrosis	DMN-induced liver damage in rats	In vivo	Reduced hepatic collagen accumulation via the inactivation of HSCs	[6]
	TGF-*β*1-treated rat HSCs	In vitro	Suppression of ECM expression through inhibition of Smad3 signaling	[87]
	High cholesterol-induced NASH in rats	In vivo	Improvement of liver oxidative and inflammatory status and reduction of hepatic collagen deposition through the downregulation of NF-*κ*B and MMP-2/9, respectively	[88]
	CCl4-induced fibrosis in rats	In vivo	Prevented CCl_4_-induced liver inflammation, necrosis, and fibrosis through suppression of oxidative stress, NF-*κ*B, TGF-*β*/Smad3, and JNK/Smad3 pathways	[89, 90]
	Alcohol-induced hepatic damage in mice	In vivo	Attenuated liver inflammation, fibrosis, and hepatocyte apoptosis via decreasing the NF-*κ*B, TGF-*β*1, and caspase-3 levels	[91]
	CCl4-induced fibrosis in mice, TGF-*β*1-treated rat HSCs	Both in vitro and vivo	Increased targeting of HSCs, ameliorated liver injury and fibrosis via SPARC-dependent pathways	[92]
	ApoE^−/−^-induced NASH in mice, mouse hepatocyte AML-12	Both in vitro and vivo	Suppressed hepatic steatosis, oxidative stress, inflammation and fibrosis through modulating hepatic SIRT1-mediated signaling cascades	[93]
	CCl4-induced fibrosis in rats	In vivo	Reduced liver fibrosis and inflammation by the upregulation of MMP-2 activity and downregulation of proinflammatory cytokines levels	[94]
Cardiac fibrosis	Pressure overload-induced cardiac remodeling in mice	In vivo	Attenuated cardiac hypertrophy and interstitial fibrosis via the inhibition of PI3K/Akt, ERK, and JNK signaling	[70]
	TGF-*β*1-treated CFs	In vitro	Inhibited CF proliferation, differentiation, and collagen synthesis via G0/G1 arrest	[98]
	Hypertension-induced atrial fibrosis in rats, hydrostatic pressure-treated CFs	Both in vitro and vivo	Alleviated the atrial fibrosis in SHRs and inhibited CF proliferation and profibrotic marker expression by inactivating Smad3 signaling	[7]
	AngII-treated CFs	In vitro	Suppressed profibrotic genes expression via inactivating Smad3 signaling	[99]
Lung fibrosis	Bleomycin-induced pulmonary fibrosis in mice	In vivo	Attenuated pulmonary fibrosis through inhibiting TGF-*β*1 secretion and decreasing regulatory T cells	[5]
	Allergen-induced chronic asthma in mice	In vivo	Inhibited airway remodeling and peribronchial fibrosis probably through reducing Th2 cytokines levels and oxidative stress	[104]
	HDM-induced chronic asthma in mice	In vivo	Improved airway inflammation and fibrosis potentially through inhibiting the expression of proinflammatory cytokines and TGF-*β*	[105]
	MP-induced pneumonia in mice, MP-treated BEAS-2B cell line	Both in vitro and vivo	Suppressed lung inflammation and fibrosis by inhibition of autophagy activation after MP infection	[106]
	Radiation-induced lung injury in rodents	In vivo	Ameliorated the lung injury including lung fibrosis by lowering IL-1*β* level and maintaining the homeostasis of inflammatory factors	[107]
Renal fibrosis	Daunorubicin-induced nephrotoxicity in rats	In vivo	Improved nephrotoxicity by reducing renal fibrosis, inflammation, and oxidative/ER stress through mitigating AT1R, ERK1/2-NF-*κ*B p65 signaling pathways	[113]
	A mouse model of UUO, TGF-*β*1-treated NRK52E cell line	Both in vitro and vivo	Relieved renal fibrosis in vitro and in vivo by blocking Smad3 signaling	[114]
	STZ-induced diabetic nephropathy in rats, high glucose-treated cell line	Both in vitro and vivo	Attenuated the deposition of ECM in vitro and in vivo and inhibited cell proliferation in vitro, through let-7a-mediated inhibition of TGF-*β*1/smad signaling	[115]
	A rat model of renovascular hypertension	In vivo	Ameliorated hypertensive renal damage, including interstitial fibrosis, by modulating the balance of components of the renin-angiotensin system	[116]
	A mouse model of lupus	In vivo	Reduced the autoimmunity and prevented kidney damage including fibrosis by modulating T-cell subsets and cytokine profile	[117]
Skin fibrosis	Mechanical stretch-induced hypertrophic scars in mice	In vivo	Attenuated skin fibrosis and inhibited scar formation via the inhibition of dermal fibroblast activation and local inflammation	[124]

Abbreviations used are DMN: dimethylnitrosamine; HSCs: hepatic stellate cells; ECM: extracellular matrix; NASH: nonalcoholic steatohepatitis; SPARC: secreted protein acidic and rich in cysteine, CFs: cardiac fibroblasts; HDM: house dust mite; MP: mycoplasma pneumonia; ER: endoplasmic reticulum; STZ: streptozotocin.
